# Analyzing the Differences in the Degree of Force Application Between Novice and Expert Physiotherapists Using a Muscle Deformation Sensor

**DOI:** 10.7759/cureus.59801

**Published:** 2024-05-07

**Authors:** Satoshi Shimabukuro, Tamon Miyake, Emi Tamaki

**Affiliations:** 1 Graduate School of Engineering and Science, The University of the Ryukyus, Nishihara, JPN; 2 Future Robotics Organization, Waseda University, Tokyo, JPN

**Keywords:** expert, novice, physiotherapy, degree of force, muscle deformation

## Abstract

Background: Training physiotherapists require substantial experience and a lengthy period of time to achieve proficiency. However, establishing an objective method for quantifying the degree of force applied during treatment remains elusive, making training difficult.

Objectives: This study aims to clarify the difference in the degree of force application between novice and expert physiotherapists using muscle deformation sensors and to assist in teaching.

Methods: A muscle deformation sensor array was utilized to capture the muscle bulging (muscle deformation), and the degree of force was visualized. The experiment involved two types of physiotherapy: upper and lower extremity exercises. Subsequently, the muscle deformation value and standard deviations of the muscle deformation data obtained were compared.

Results: Significant differences between novices and experts were observed in forearm muscle deformation values and standard deviations across both types of physiotherapies (p<0.05). Additionally, a distinction was observed in the left lower limb flexor muscles during upper extremity exercise (p<0.05).

Conclusion: The results of this survey showed notable differences in the degree of force application between novices and experts, as demonstrated by our findings. Moreover, these implications extend beyond physiotherapy to sports, hobbies, and the teaching of traditional skills.

## Introduction

Physiotherapy techniques encompass a wide range of procedures administered by physiotherapists to aid recovery, enhance functional ability, reduce pain, and improve quality of life. Manipulation of soft tissues and joints is also commonly required to improve mobility and alleviate discomfort. Each technique is tailored to the specific needs and conditions of the patient to optimize recovery. Physiotherapy emphasizes the importance of manual dexterity and body mechanics in executing procedures, necessitating extensive training and experience for proficiency. Typically, a decade of practice is required to achieve expertise in the field [1]. To address these challenges, wearable devices have been developed for visualizing and quantifying movement analysis [2-4] as well as for visualizing plantar pressure [5]. Studies have also examined muscle strength using force plates, revealing that physiotherapy students often exhibit lower hand strength during physiotherapy [6,7]. Other studies have compared the distance traveled by the center of foot pressure when weight transfer was induced in physiotherapists. The results of this study reported that there was no difference in the distance traveled by the center of foot pressure for those with more than six years of experience [8].

However, much remains unknown about the level of force applied during patient manipulation, particularly regarding superficial and deep touch sensations. While methods have been proposed to detect stress and strain in superficial senses [9] and provide tactile feedback [10], real-time measurement of deep sensory force and strain is lacking. Consequently, the extent of differences between novice and expert physiotherapists in this regard is still being determined.

Observations suggest that the quality of treatment tends to vary more among novices than experts, with experts providing excellent and consistent care with high efficiency [11]. While novices often experience greater post-treatment fatigue and stress due to patient volume and workload [12], experts manage fatigue more effectively and can perform many procedures in a day. Building upon these observations, we proposed two hypotheses regarding differences in deep sensory power between novices and experts: (1) Experts apply less unnecessary force, resulting in lower overall force application during treatment compared to novices. (2) Experts show less variability in force application during treatment compared to novices.

Experiments were conducted to verify those hypotheses. Thus, this study aims to utilize muscle deformation sensor arrays to visualize muscle conditions and determine the differences in the degree of force application between novices and expert physiotherapists.

## Materials and methods

Participants

Novice and expert physiotherapists were recruited for this study (Table 1).

**Table 1:** Participants’ characteristics

		Novice (n=10)	Expert (n=10)	p-value
Age (years old)		19 ± 2.2	39.2 ± 5.2	0.0001
Sex (male/female)		7/3	9/1	0.5820
Height (cm)		163.3 ± 9.5	167.6 ± 6.5	0.2564
Body weight (kg)		57.5 ± 9.2	67.2 ± 12.8	0.0680
Forearm length (cm)	Left	25.1 ± 2.1	26.6 ± 1.1	0.0707
	Right	25.3 ± 2.1	26.5 ± 1.2	0.1173
Lower leg length (cm)	Left	36.3 ± 5.0	37.7 ± 5.8	0.1704
	Right	36.5 ± 4.4	37.5 ± 6.1	0.1836
Forearm circumference (cm)	Left	23.1 ± 5.8	24.4 ± 5.4	0.1102
	Right	23.2 ± 5.7	25.2 ± 5.4	0.0942
Lower leg circumference (cm)	Left	33.5 ± 2.7	32.9 ± 3.3	0.6892
	Right	33.6 ± 2.7	33.3 ± 3.3	0.8261
Experience (years)		-	15.3 ± 3.7	-

The study subjects were participants whose consent was obtained after the implementer gave them oral and written explanations.

The inclusion criteria for novice students were individuals with no basic knowledge and practice experience in physiotherapy and less than one month of work experience in a medical care setting. Ten first-year students from a three-year physiotherapy training program met these criteria and participated as novices in this study. The inclusion criteria for experts were individuals licensed as physiotherapists with a minimum of 10 years of experience, a history of teaching at physiotherapy training schools, and a degree or certification in physiotherapy specialization. Expert selection depended on the three core elements of physiotherapy: clinical practice, education, and research [13]. Thus, 10 physiotherapists who met these criteria were selected as experts in this study. Additionally, all participants were required to meet the following conditions: (a) They must have completed medical treatment and rehabilitation and must not regularly use prescribed medications. (b) They must not have any significant pre-existing medical conditions. (c) Participants should be in good physical condition on the day of the experiment, without injuries or muscle soreness that could interfere with exercise. (e) Additionally, they should have had at least seven hours of sleep and abstained from alcohol the day before or on the day of the experiment. Exclusion criteria included any conditions that could interfere with the experiment or require special considerations (Table 2).

**Table 2:** Exclusion criteria

Questions								
1. Do you have a pre-existing or serious medical condition, such as heart disease or illness?								
2. Do you regularly use medicines prescribed by a doctor?								
3. Do you have an injury or illness for which treatment and rehabilitation have not been completed?								
4. Are you pregnant or possibly pregnant?								
5. Do you have skin atopy?								
6. Have you been diagnosed by a doctor as anemic?								
7. Other cases where the practitioner considers the experiment unsuitable.								
8. Are you unwell?								
9. Do you have any injuries or muscle pain affecting physical exercise?								
10. Do you sleep less than 7 hours during the day?								
11. Have you consumed alcohol today or the day before?								

Equipment

**Figure 1:**
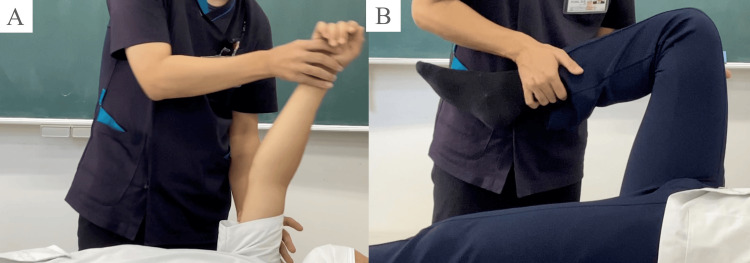
Range of motion for experimental tasks (A) Upper extremity raising exercise: The starting position of the experiment was set with the elbow joint of the human model extended and in contact with the bed. The reached position was when the human model’s upper limb moved to a point where the shoulder joint reached 90°. The end position was set in the same way as the start position. (B) Lower extremity flexion exercise: The experiment’s starting position was where the lower limb of the human model was in contact with the bed in an extended position. The position reached was 90° hip flexion. The end position was set the same as the start position. The participant was instructed to position their lower limbs comfortably for the task. Image Credit: Author

Participant recruitment and the experimental period occurred between May 1, 2022, and September 30, 2022. The experiment included two defined exercises, "upper extremity raising exercise" and "lower extremity flexion exercise." During the experiment, participants performed these tasks altruistically using a human body model for nursing practice (Figure 1).

The tasks were defined by their starting, ending, and reached positions.

Upper Extremity Raising Exercise

The starting position of the experiment was set with the elbow joint of the human model extended and in contact with the bed. The arrival position was set at 90° shoulder joint flexion. The end position of the movement was the same as the start position. Participants were instructed to grasp the following parts of the human model: the proximal shoulder joint with the left hand from the ventral side and the distal forearm with the right hand from the dorsal side. They were also instructed to position their lower extremities comfortably for the task.

Lower Extremity Flexion Exercise

The starting position was where the lower extremity of the human model was in contact with the bed in an extended position. The position reached was 90° hip flexion, and the end position was the same as the start position. Participants were instructed to grasp the thigh of the human model with the left hand from the dorsal side and the distal shank with the right hand from the ventral side. The position of the participant's lower extremities remained the same as in the upper extremity raising exercise. One researcher conducted the experiment.

Task Procedure

Step 1: Environmental adjustment: To minimize external influence and standardize the experiment [14], the positions of both the human body model and the participants were adjusted. Prior to commencing the experimental task, participants aligned their lumbar position with the pelvic girdle of the human model. They were instructed to wear shoes during the experiment. Vertical bars were positioned to indicate specific positions reached. Tape markers, measuring 4.5 cm in length and 2 cm in width, were placed on the middle finger of the right hand and on the front surface of the right thigh of the human model.

Step 2: Confirmation of mounting position: The device's position on the participants was determined by measuring the forearm and lower leg lengths. The device was positioned at the center of each measured length. Forearm length was defined as the distance from the lateral epicondyle of the humerus to the radial styloid process, while lower leg length was defined as the distance from the patellar fissure to the lower end of the lateral malleolus.

Step 3: Experimental procedures: Participants were briefed on the experimental task and practiced it 10 times without any guidance or feedback that could influence their performance during the actual experiment. Afterward, the experimental task was conducted 10 times, with a break after the fifth attempt. For each task, the equipment was first worn on the right forearm, the left forearm, the right lower leg, and the left lower leg.

Equipment Used for Measurements

**Figure 2:**
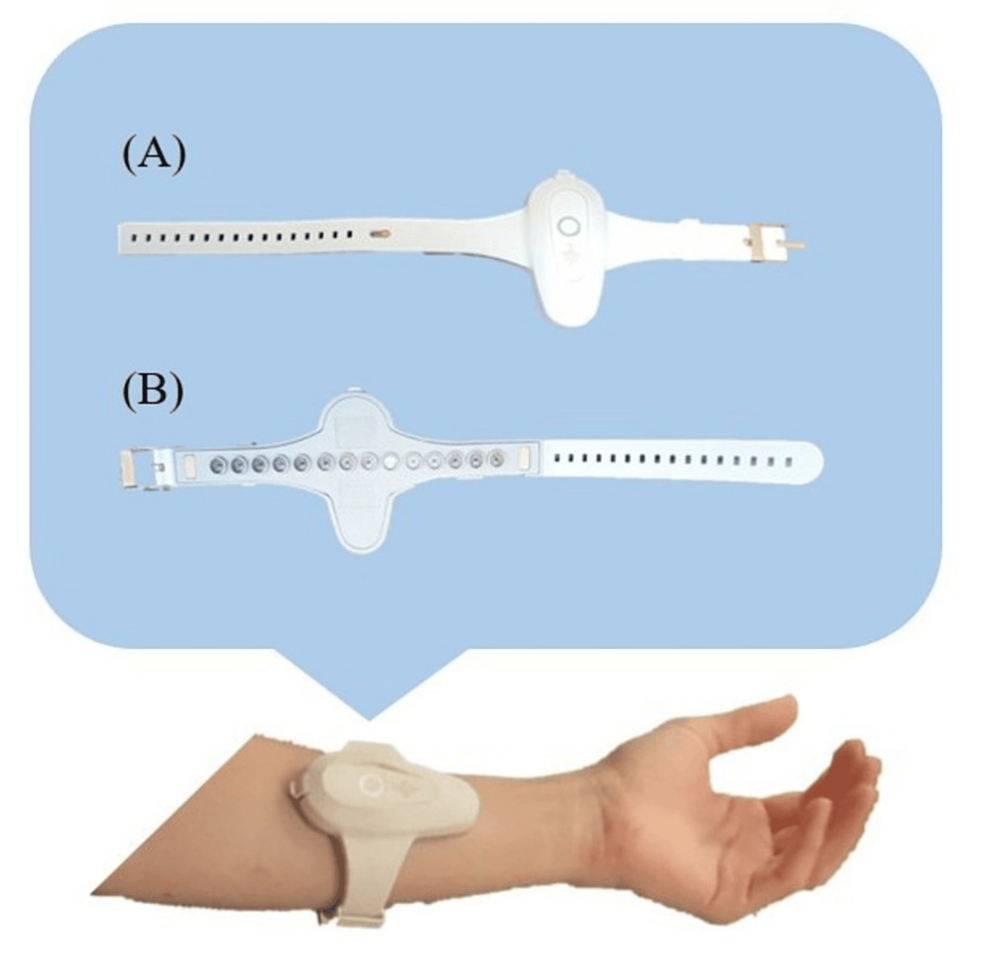
Equipment used for measurements (A) Surface. (B) Sensor surface. The muscle deformation sensor array has 14 channels of optical muscle deformation sensors, as well as a gyro sensor and a three-axis acceleration sensor. Image Credit: Author

This study used a muscle deformation sensor array, FirstVR (H2L Inc., Tokyo, Japan), as shown in Figure 2 [15,16].

The sensor array comprises 14 optical muscle deformation sensors capable of measuring muscle bulge (muscle deformation) and estimating intrinsic sensation. Additionally, the device has a gyro sensor and a three-axis acceleration sensor, making it possible to acquire quaternion data (posture data of the body part wearing the device). It can be worn around the forearm like a wristwatch to measure muscle deformation in the forearm and fingers [17] or on the lower leg to measure muscle deformation in the lower leg muscles.

Pretreatment Data

**Figure 3:**
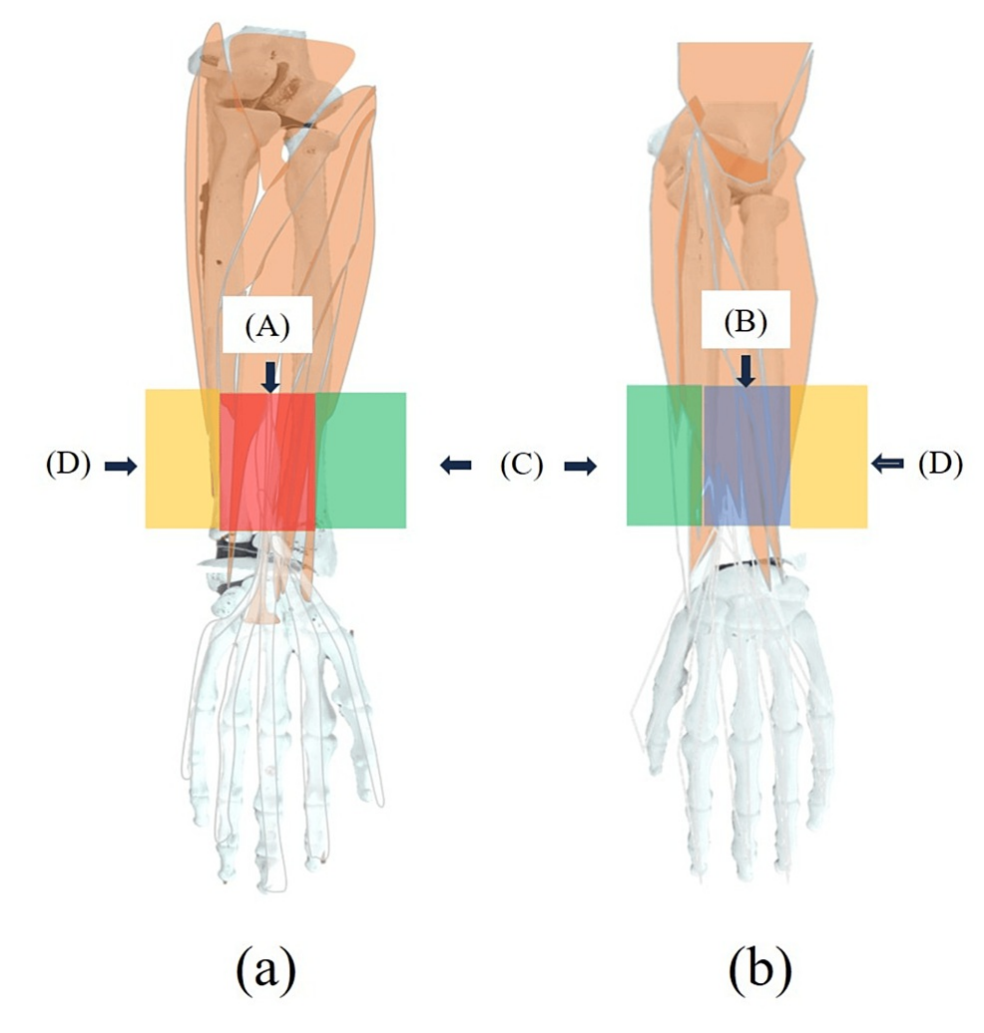
Classification of forearm muscle deformation sensors The muscles of the forearm were defined as (A) flexor muscles, (B) extensor muscles, (C) thumb side muscles, and (D) little finger side muscles. The palm flexors and finger joint flexors of the wrist joint were considered A, and the dorsiflexors and finger joint extensors of the wrist joint were considered B. The brachioradialis, radial carpal flexors, and radial carpal extensors were defined as C, and the ulnar carpal flexors and ulnar carpal extensors were defined as D. Image Credit: Author

**Figure 4:**
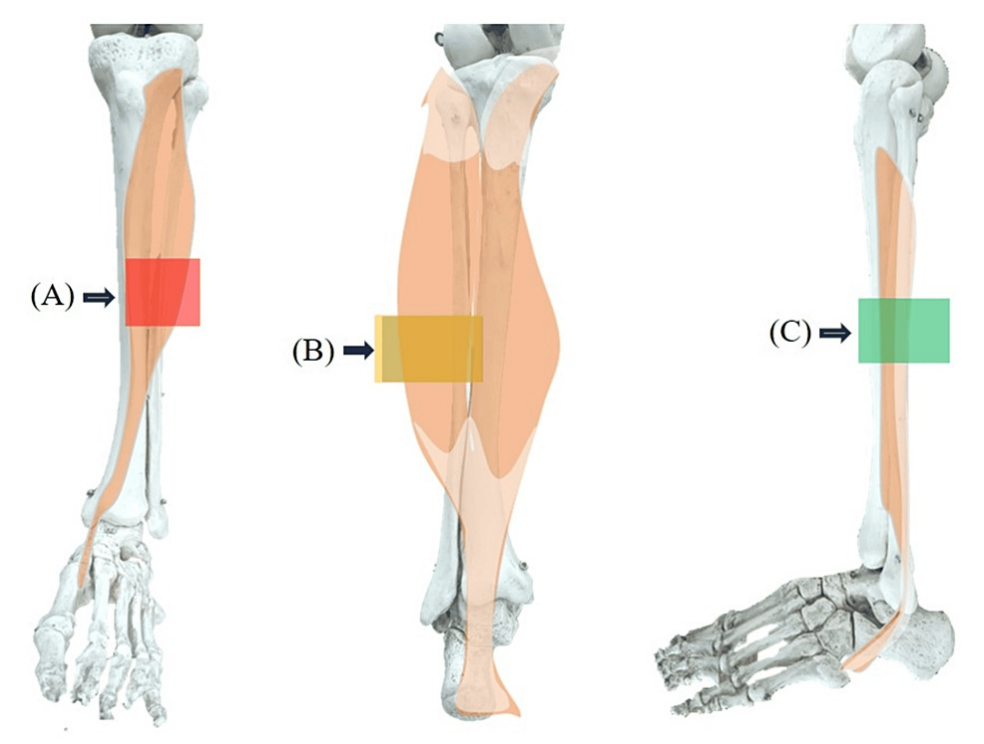
Classification of lower leg muscle deformation sensors In the lower leg, (A) the tibialis anterior muscle was the extensor, and (B) the lateral head of the gastrocnemius muscle was used as the flexor. (C) The peroneal muscle was also designated as the lateral muscle. Image Credit: Author

The start and end positions of the experiment were determined based on the quaternion data. Muscle deformation data obtained from the sensor array underwent moving average processing. Forearm and lower leg muscle deformation data were categorized as follows: The forearm was classified into the flexor, extensor, thumb side muscle, and little finger side muscles (Figure 3), whereas the lower leg was categorized into the flexor, extensor, and lateral muscles (Figure 4).

Statistical analysis

Data acquired from the 10 trials were averaged for analysis. Novices and experts were compared in terms of the sum of the maximum and minimum muscle deformation data and the standard deviation of muscle deformation. Statistical analysis was conducted using R version 4.2.2 software (The R Foundation, Indiana, USA), with power set at 0.6, effect size (delta) at 0.8, and significance level at 0.05. The number of samples (n) was set at 10, meaning there were 10 novices and 10 experts. The Shapiro-Wilk test was initially used to assess the normality of comparison data, followed by the independent t-test or Mann-Whitney U-test. The significance level was set at 0.05.

Ethical considerations

This study was conducted in accordance with the Declaration of Helsinki. It was also approved by the Ethical Review Committee of The Okinawa Rehabilitation Welfare Institute (approval number: 2021-04). Participants were orally briefed on the study details with the documented content provided. For student participants, it was clarified that their academic performance would not be affected whether they chose to participate or not. Consent from parents or guardians of minors was waived by the Ethical Review Committee.

## Results

**Figure 5:**
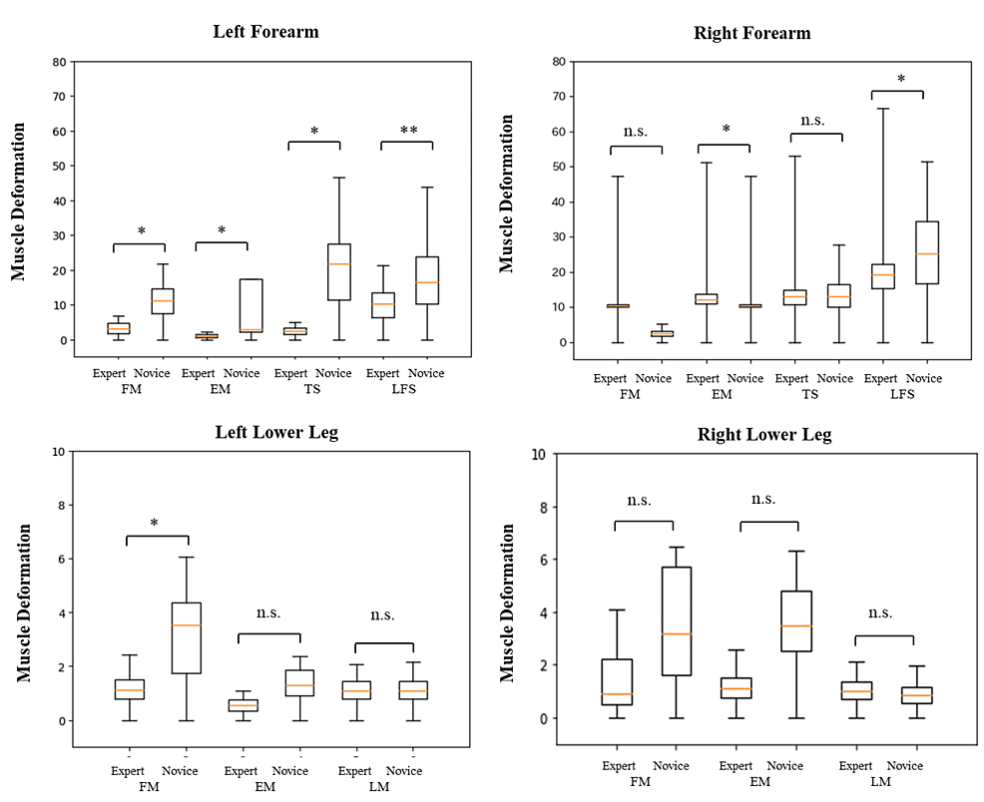
Muscle deformation at various sites during upper extremity raising exercises Significant differences were found in muscle deformation in the flexor and extensor muscles of the left forearm (p<0.05), on the thumb side (p<0.05) and little finger side (p<0.01) of the left forearm, and on the little finger side of the right forearm (p<0.05). Notably, in the lower leg, differences were found in the left flexor (p<0.05). * p<0.05, ** p<.001 n.s.: not significant, FM: flexor muscle, EM: extensor muscle, TS: thumb side muscle, LFS: little finger side muscle, LM: lateral muscle Image Credit: Author

**Figure 6:**
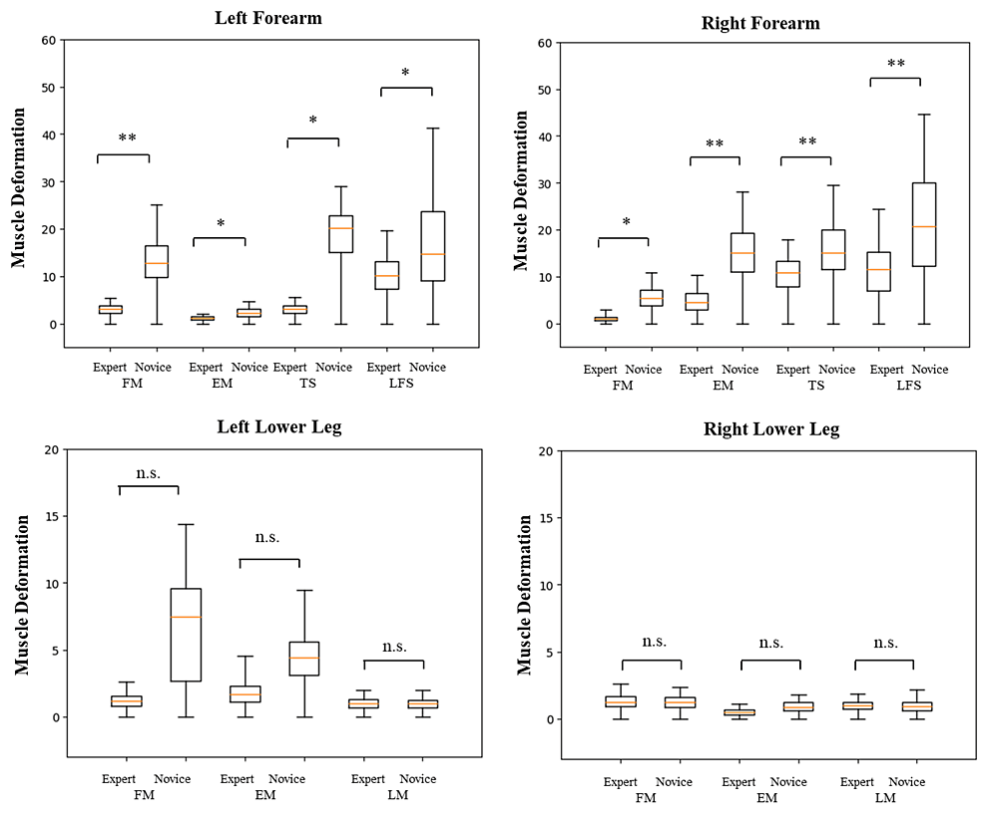
Muscle deformation at various sites during lower extremity flexion exercises Significant differences in muscle deformation were found across all muscles of both forearms (p<0.05). Specifically, differences were found in the flexor muscles of the left forearm and the extensor muscles of the right forearm, thumb, and little finger (p<0.01). However, no significant differences were observed between the lower leg muscles. Nonetheless, there was a trend toward differences between participants in the extensor muscles (p=0.07) and the lateral muscles (p=0.05) of the left lower leg. * p<0.05, ** p<0.01 n.s.: not significant, FM: flexor muscle, EM: extensor muscle, TS: thumb side muscle, LFS: little finger side muscle, LM: lateral muscle Image Credit: Author

The degree of force applied during the upper extremity raising and lower extremity flexion exercises remarkably differed between novices and experts. Tables 3-4, along with Figures 5-6, illustrate this difference, with experts consistently exhibiting smaller values of muscle deformation and variation compared to novices. This discrepancy was particularly noticeable on the thumb and the little finger sides of the forearm.

**Table 3:** Muscle deformation and standard deviations at various sites during upper extremity raising exercises MD: muscle deformation, SD: standard deviation, FM: flexor muscle, EM: extensor muscle, TS: thumb side muscle, LFS: little finger side muscle, LM: lateral muscle

		Novice (n=10)		Expert (n=10)		MD	SD
		Mean ± SD		Mean ± SD		p-value	p-value
Left forearm	FM	101.4 ± 1.8		93.9 ± 0.5		0.0244	0.1051
	EM	133.8 ± 4.9		103.5 ± 1.8		0.0142	0.0068
	TS	126.7 ± 9.7		101.9 ± 1.1		0.0400	0.0524
	LFS	126.1 ± 8.5		91.4 ± 4.7		0.0003	0.0021
Right forearm	FM	101.3 ± 1.0		92.8 ± 1.3		0.2581	0.8534
	EM	132.3 ± 5.6		101.8 ± 2.5		0.0939	0.1230
	TS	124.9 ± 5.2		94.6 ± 3.3		0.1135	0.1431
	LFS	121.0 ± 12		95.5 ± 5.6		0.0400	0.0232
Left lower leg	FM	102.8 ± 2.1		83.9 ± 1.1		0.0321	0.6305
	EM	113.7 ± 1.5		112.6 ± 0.6		0.9330	0.1903
	LM	88.0 ± 0.4		85.3 ± 0.4		0.0912	0.3527
Right lower leg	FM	123.1 ± 1.6		110.6 ± 0.5		0.1050	0.3930
	EM	87.4 ± 0.6		82.7 ± 0.3		0.1050	0.5290
	LM	85.7 ± 0.4		83.9 ± 0.4		0.1530	0.7810

**Table 4:** Muscle deformation and standard deviations at various sites during lower extremity flexion exercises MD: muscle deformation, SD: standard deviation, FM: flexor muscle, EM: extensor muscle, TS: thumb side muscle, LFS: little finger side muscle, LM: lateral muscle

		Novice (n=10)		Expert (n=10)		MD	SD
		Mean ± SD		Mean ± SD		p-value	p-value
Left forearm	FM	130.5 ± 5.1		102.3 ±1.1		0.0244	0.1051
	EM	100.5 ± 1.0		93.7 ± 0.4		0.0142	0.0068
	TS	128.2 ± 6.3		102.0 ± 1.0		0.0400	0.0524
	LFS	110.5 ± 9.7		97.4 ± 4.1		0.0003	0.0021
Right forearm	FM	102.9 ± 2.4		92.4 ± 0.6		0.2581	0.8534
	EM	121.7 ± 5.9		95.1 ± 2.2		0.0939	0.1230
	TS	120.5 ± 6.3		99.9 ± 3.6		0.1135	0.1431
	LFS	117.0 ± 10.9		86.5 ± 5.6		0.0400	0.0232
Left lower leg	FM	99.4 ± 4.5		89.8 ± 0.5		0.0321	0.6305
	EM	117.7 ± 1.9		114.0 ± 0.9		0.9330	0.1903
	LM	89.0 ± 0.4		85.1 ± 0.4		0.0912	0.3527
Right lower leg	FM	121.9 ± 2.1		112.2 ± 0.5		0.1050	0.3930
	EM	86.4 ± 0.4		84.0 ± 0.4		0.1050	0.5290
	LM	86.0 ± 0.4		83.1 ± 0.3		0.1530	0.7810

Characteristics of the participants

Table 1 presents the characteristics of the participants. Novices had an average age of 19.0 ± 2.2 years, whereas experts were significantly older, with an average age of 39.2 ± 5.2 years (p<0.01). Experts had an average of 15.3 ± 3.7 years of experience, and there were no significant differences in gender, height, weight, forearm and lower leg length, or forearm and lower leg circumference between the two groups.

Total muscle deformation

Experts exhibited lower total muscle deformation than novices across all upper extremity raising and lower extremity flexion exercises. In the upper extremity raising exercises, differences were observed in the flexor (p<0.05) and extensor (p<0.05) muscles of the left forearm of novices and experts, thumb side (p<0.05), and little finger (p<0.01) of the forearm. Differences were also noted on the little finger side of the right forearm (p<0.05) and in the flexor muscles of the left lower leg (p<0.05). However, no differences were found in the force exertion of the right forearm flexor, extensor, and matrices, the left lower leg extensor and lateral muscles, and the right lower leg flexor, extensor, and lateral muscles (Table 3, Figure 5). In the lower extremity flexion exercise, differences were observed in the flexor, extensor, thumb side, and the little finger muscles of both the right and left forearms between novices and experts (p<0.01). Notably, no differences were found in the flexor, extensor, and lateral muscles of both the left and right lower legs. However, a trend toward differences was observed in the left lower leg extensor (p=0.07) and lateral (p=0.05) muscles (Table 4, Figure 6).

The standard deviation of muscle deformation values

The standard deviation of muscle deformation values was consistently lower for experts compared to novices during upper extremity raising and lower extremity flexion exercises. Significant differences between novices and experts were observed in upper extremity raising exercises, specifically in the extensor (p<0.01) and little finger side of the left forearm (p<0.01), as well as the little finger side of the right forearm (p<0.05). However, no differences were found in the flexor, thumb side, and muscles of the left forearm; the flexor, extensor, and thumb side muscles of the right forearm; and the flexor, extensor, and lateral muscles of the left and right lower legs (Table 3, Figure 5). During the forearm measurements in the lower extremity flexion exercise, differences were noted in the flexor (p<0.05), extensor (p<0.05), and thumb (p<0.05) and little finger (p<0.05) sides of both the right and left forearms between novices and experts. Particularly, significant differences were observed in the flexor and little finger sides of the forearm and the thumb side of the forearm (p<0.01). In the lower leg, no differences were found in the flexor, extensor, and lateral muscles of both the left and right sides (Table 4, Figure 6).

## Discussion

Characteristics of the participants

This study revealed a difference in years of experience between novices and experts, which arose as a result of defining an expert as someone with more than 10 years of experience. While age differences among participants may raise concerns about their impact on physical function [18], it is worth noting that many of the differences in physical function are typically observed in individuals aged 65 years and older [19]. However, this age difference did not significantly affect the outcomes of the present study.

Total muscle deformation

Expert physiotherapists typically classify forces below 1 N as "light touch" [20-22]. Consequently, the lower muscle deformation values observed in this study could be attributed to experts' heightened awareness of force levels and their continuous refinement of technique. Upon regional examination, differences in muscle deformation were noted across all forearm forces (p<0.05). Experts skillfully adjusted the flexor and extensor muscles of the forearm and controlled the force levels on the thumb and little finger sides. Significant differences were observed in the flexor muscles of the left lower leg during the lower extremity flexion exercise and in the extensor (p=0.07) and lateral muscles (p=0.05) of the left lower leg during the lower leg flexion exercise, suggesting differences between novices and experts. The lower leg flexors control the upward weight transfer in the body, while the lower leg extensors and lateral muscles control the lateral weight transfer. Novices speculated that the reason for the larger muscle deformation values was their unfamiliarity with controlling weight transfer during the exercise and difficulty in adjusting the force levels. In contrast, experts minimized the muscle deformation on the side of the weight shift, facilitating the weight transfer process.

The standard deviation of muscle deformation values

In this study, we hypothesized that novices would have difficulty adjusting their forces, resulting in greater fluctuations in hand and leg forces than skilled performers. The expert performers showed less variation in force adjustment and differed from novice performers (p<0.05). In grasping movements, skilled performers are known to predict the weight of an object in advance [23], suggesting that they are skilled in predicting and adjusting force even before starting the grasping movement. In addition, it was shown that skilled users show the ability to quickly correct the force level when necessary, while novice users may need assistance in predicting and adjusting the force level.

Future prospects

This study revealed a noticeable difference in the degree of force exerted between novices and experts. Novices typically acquire new skills through repeated trials and errors. Moreover, since supervised learning and visual instruction have been deemed effective in facilitating learning among novices [24,25], there is a need to establish a teaching method that analyzes and visualizes experts’ data. Software applications capable of visualizing and instructing on expert muscle deformation data are predicted to be effective [26], especially within augmented reality and virtual reality environments [27,28], as these applications hold promising potential for enhancing novice learning and skill acquisition.

Study limitations

For experts, physiotherapy techniques for a range of motion exercises are refined over years of experience. Therefore, given that grasping positions vary among individuals, the influence of specifying the hand's holding position should be acknowledged. Furthermore, numerous joint range-of-motion exercises exist for major large joints [29]. Therefore, this examination needs to be extended to other range of motion exercises, as only two tasks were examined in this study. It is also possible that the situation of a novice who does not have any specialized knowledge of physiotherapy may have had a small influence on this comparison of force levels.

## Conclusions

This study aims to assess muscle conditions during physiotherapy and identify differences in the degree of force applied between novices and expert physiotherapists. Utilizing a muscle deformation sensor array capable of detecting and visualizing muscle deformation, two distinct physiotherapy techniques were performed, and the muscle deformations and standard deviations obtained during these techniques (n=10) were compared. Significant differences were observed between novices and experts in terms of muscle deformation and standard deviation for the right forearm (p<0.05) and muscle deformation for the left forearm (p<0.05). Notably, during the upper extremity raising exercise, significant differences were observed in the flexor muscles of the left lower leg (p<0.05). These findings underscore the difference in the degree of force application between novice and experienced practitioners, particularly highlighting differences in the force distribution on the thumb and little finger sides of the forearm. Building on these findings, a system emphasizing the "degree of force on the thumb and little finger side of the forearm" is envisaged, which will facilitate efficient and independent learning for novice physiotherapists.
